# A Case of Neonate with Split Cord Malformation Presenting with Hypoplasia of the Left Lower Extremity

**DOI:** 10.3390/medicina59040726

**Published:** 2023-04-07

**Authors:** Ryosuke Tanimoto, Tamaki Ikuse, Natsuki Ito, Hiroyuki Sato, Yuriha Kasai, Hiromichi Yamada, Nobutomo Saito, Tomohiro Iwasaki, Mitsuru Ikeno, Hiroki Suganuma, Ken Hisata, Hiromichi Shoji, Takahiro Kudo, Koichiro Sakamoto, Kazuaki Shimoji, Akihide Kondo, Toshiaki Shimizu

**Affiliations:** 1 Department of Pediatrics, Juntendo University Faculty of Medicine, 3-1-3 Hongo, Bunkyo, Tokyo 113-8431, Japan; 2 Department of Neurosurgery, Juntendo University Faculty of Medicine, 3-1-3 Hongo, Bunkyo, Tokyo 113-8431, Japan; 3 Department of Neurosurgery, International University of Health and Welfare Narita Hospital, 852 Hatakeda Narita, Chiba 286-0124, Japan

**Keywords:** split cord malformation, leg hypoplasia, leg atrophy, lumbosacral abnormality, lower extremity

## Abstract

The frequency of split cord malformation (SCM) is approximately 1 in 5000 births; however, patients are rarely diagnosed with SCM in the neonatal period. Moreover, there have been no reports of SCM with hypoplasia of the lower extremities at birth. A 3-day-old girl was transferred to our hospital for a thorough examination of hypoplasia of the left lower extremity and lumbosacral abnormalities detected after birth. The spinal magnetic resonance imaging (MRI) revealed a split spinal cord in a single dural tube. Based on the MRI findings, the patient was diagnosed with SCM type II. Following discussions with the parents, pediatricians, neurosurgeons, psychologists, and social workers, we decided to perform untethering to prevent further neurological impairment after achieving a sufficient body weight. The patient was discharged on day 25 of life. Early diagnosis and intervention may improve the neurological prognosis in terms of motor function, bladder and bowel function, and superficial sensation; thus, clinicians should report infrequent findings that may lead to SCM diagnosis. SCM should be differentiated in patients with left–right differences in the appearance of the lower extremity, particularly in those with lumbosacral abnormalities.

## 1. Introduction

Split cord malformation (SCM) is a congenital anomaly that results from a developmental anomaly of the notochord and is anatomically classified into type I and type II subtypes. In type I SCM, the spinal cord is divided by a bony or cartilaginous septum into two dural tubes, each with its own spinal cord. Type II SCM presents with two spinal cords separated by a fibrous septum in a single dural tube [1]. Mahapatra et al. proposed a new subclassification of type I SCM based on the intraoperative location of a bone spur causing the split, which may have a bearing on surgical dissection and outcome [2]. Based on the authors’ experience with 25 cases of type I SCM, they have classified the disorder into four subtypes: Type Ia, bone spur located in the center with duplicated cord above and below the spur; Type Ib, bone spur at the superior pole with no space above it; Type Ic, bone spur at the lower pole with large duplicated cord above; and Type Id, bone spur straddling the bifurcation with no space above or below the spur. SCM is associated with abnormal findings in the lumbosacral region, spinal meningocele, scoliosis, and dysplasia of the spine and ribs [3]; nonetheless, clinicians have not reported on hypoplasia of the lower extremities in neonates. In this report, we describe our experience with a neonate diagnosed with type II SCM based on a thorough examination of hypoplasia of the left lower extremity and lumbosacral abnormalities observed at birth.

## 2. Case Report

**Figure 1:**
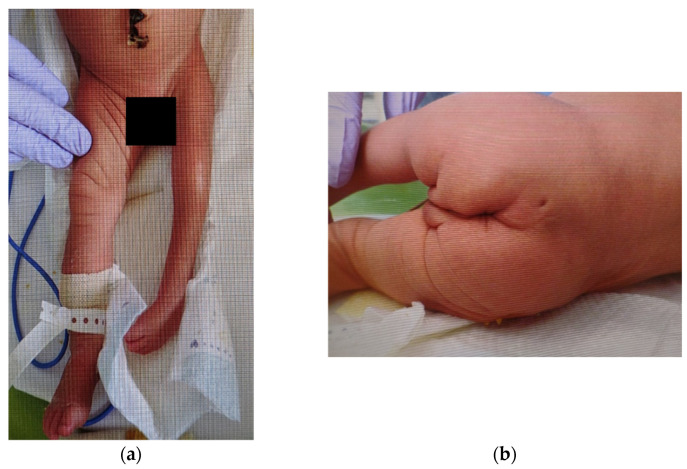
External findings of the lower extremities and buttocks: (**a**) Hypoplasia is observed in the left lower extremity. Spontaneous movements and tendon reflexes are absent. There are no abnormal findings in the right lower extremity; (**b**) A small dimple is observed in the skin of the lumbosacral region and a curvature of the gluteal cleft.

A 3-day-old girl presented with hypoplasia of the left lower extremity and lumbosacral skin abnormalities at birth. The patient was the second child of non-consanguineous parents without a family history of deformity of the extremities or any neurological disorders. Following a spontaneous pregnancy, her mother underwent a prenatal checkup at another hospital. There were no abnormalities during pregnancy, and the fetal ultrasonography did not indicate any abnormalities in the morphology of the lower limbs or spine. Her mother had no history of fertility treatment, alcohol consumption, or smoking. Since the mother had a history of cesarean section, the patient was delivered by a scheduled cesarean section at 38 weeks and 1 day. Her respiratory status was stable at birth; she did not require resuscitation. The Apgar scores were 8 points and 9 points for 1 min and 5 min, respectively. The patient had a birth weight of 2920 g (+0.29 standard deviation), a height of 46 cm (−1.08 standard deviation), and a head circumference of 35.7 cm (+2.02 standard deviation). A post-natal examination revealed skin puckering in the lumbosacral region, hypoplasia, and paralysis from the left buttock to the lower extremities (Figure 1a,b). On day 3 of life, the patient was transferred to our hospital for a thorough examination of these anomalies.

**Figure 2:**
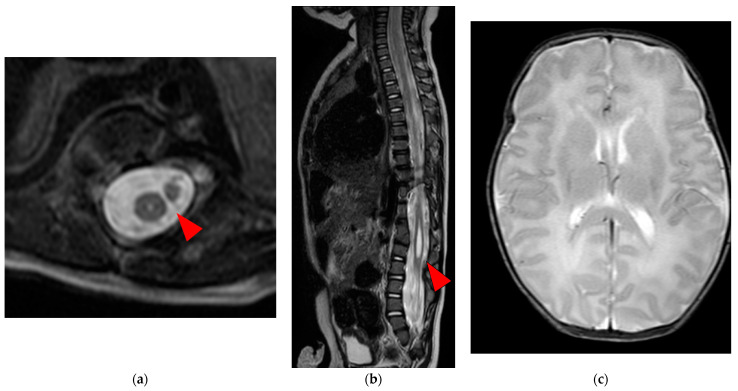
Spinal and brain MRI T2W image: (**a**) The axial section below the 10th thoracic vertebra displaying the separated spinal cord within a single dural sheath and syringomyelia (red arrow); (**b**) The sagittal section below the 10th thoracic vertebra displaying the syringomyelia (red arrow); (**c**) The axial view of the brain presented no gross abnormalities; MRI, magnetic resonance imaging; T2W, T2 weighted.

Clinical examination revealed hypoplasia (shortening and narrowing) of the left lower extremity, without any spontaneous movements or tendon reflexes. Furthermore, lumbar skin dimples, curvature of the gluteal cleft, and scoliosis were observed. Neurological findings in the right lower extremity and upper extremities were normal. There was no observable bladder–bowel disorder. Ultrasonography did not reveal abnormal findings in the intracranial space, such as hydrocephalus, intraventricular hemorrhage, and intra-abdominal space; there was no congenital heart disease. Radiographs revealed agglutination of the 9th and 10th ribs, spinal dysplasia between the 10th thoracic vertebra and sacral vertebra, scoliosis, and hypoplasia of the left lower extremity. Computed tomography revealed dysplasia of the left side of the spine below the 10th thoracic vertebra. Magnetic resonance imaging revealed a separated spinal cord within a single dural tube below the 10th thoracic vertebra, syringomyelia of the right spinal cord, left lateral deviation of the spinal cord below the second lumbar vertebra, spinal cord tethered to the left dorsally below the third lumbar vertebra, and a subcutaneous mass contiguous to the dural tube below the fourth lumbar vertebra (Figure 2). Based on the imaging findings, the patient was diagnosed with type II SCM, syringomyelia, agglutination of the ribs, vertebral malformation, scoliosis, and hemiparesis of the left lower extremity. The affected lower extremity was devoid of any reflexes or movements; therefore, we determined that the neurologic findings would not improve with untethering. Following discussions with the parents, pediatricians, neurosurgeons, psychologists, and social workers, we decided to perform untethering to prevent further neurological impairment after achieving a sufficient body weight. The patient was discharged on day 25 of life.

**Figure 3:**
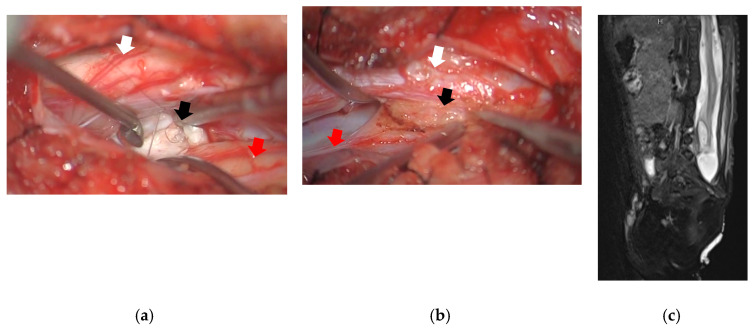
Surgical image and spinal MRI T2W image after surgery: (**a**) Surgical image at the rostral side of the split cord. There was a dermoid (black arrow) located at the ventral side of the atrophic right spinal cord (red arrow). Left spinal cord (white arrow) was not atrophic; (**b**) Surgical image at the caudal side of the split cord. There was a lipoma (black arrow) located at the caudal side of the atrophic right spinal cord (red arrow) attached to the left spinal cord (white arrow); (**c**) The sagittal view of spinal MRI showed the untethered lipoma and the improvement of the syrinx; MRI, magnetic resonance imaging; T2W, T2 weighted.

In order to avoid the development of any other symptoms on the right lower extremities by tethering of the cord, the patient underwent surgery at 10 months of age. The skin incision was created from the 10th thoracic vertebra level to the lumbosacral lipoma. Since the tethered second cord was located on the left side, laminoplasty was conducted on the left side from the 10th thoracic vertebra level to the 5th lumber vertebra level to spare the paravertebral muscles. A lipoma was seen at the caudal side of the split atrophic right spinal cord. Additionally, there was a dermoid at the rostral ventral area and the caudal area attached to the right spinal cord. Untethering was conducted at the caudal lipoma and the dermoid was removed (Figure 3).

At 3 years of age, the patient has normal motor development of the right lower extremity and is able to ambulate with the prosthesis, and there was no observable bladder–bowel disorder for the patient. Continuous evaluation for the developmental status and neurological findings, as well as rehabilitation, would be necessary for the patient. The parents provided informed consent for the publication of this case report.

## 3. Discussion

SCM originates from the formation of an accessory neuromeric canal during the embryonic period, which results in the regional splitting of the notochord and overlying neural plate (unified theory) [4]. The split portion of the cord spans several vertebral levels; however, the majority of the median septa are observed between the first and fifth levels of the lumbar vertebra. The incidence of SCM is approximately 1 in 5000 live births; however, SCM is seldom diagnosed in the neonatal period [5]. Although cases of open spina bifida, abnormal lumbosacral skin findings, and paralysis of the lower extremities may lead to the diagnosis of SCM during a thorough examination with imaging, it is difficult to diagnose cases without neurological abnormalities or a higher level of split cord. Therefore, SCM is often diagnosed in adulthood, owing to sensory deficits in the lower extremities, motor deficits, or back pain [1]. Numerous SCMs are associated with abnormal findings in lumbosacral skin lesions, including capillary hemangiomas and dermal sinuses [2]. Particularly, it is important to suspect the presence of SCM upon observing hypertrichosis of the lumbosacral region, which is reportedly present in 56% of the SCM cases [2]. There have been reports of cases with developing hypoplasia of the lower extremities due to SCM [6]; however, this patient presented with a novel symptom of hypoplasia of the lower extremities at birth. In this case, hypoplasia of the lower limbs was likely caused by disuse atrophy; nonetheless, the exact cause is unclear. Despite reports on certain cases of terminal myelocystocele with hypoplasia of the lower extremities, the mechanism by which hypoplasia of the lower extremities occurs in this disease remains unknown. In some cases of terminal myelocystocele with hypoplasia of the lower extremities, a patient could walk with the use of an orthosis [7].

The majority of neurological symptoms observed in SCM, such as motor sensory deficits and bladder–bowel dysfunction, are caused by tethered cord syndrome [3,8]. This is attributed to the association between SCM and the lesion that leads to tethering, such as thick or tight terminal filum, intradural lipoma, dermoid or dermal sinus tract, neurenteric cysts, or myelomeningoceles manqué [2]. Surgical intervention is necessary to reduce the progression of growth-associated symptoms before the onset of tethered cord syndrome with SCM [3,8]. Researchers have reported on improved neurological prognosis with surgical intervention during the asymptomatic period or immediately following symptom onset [3,8]. However, it is unknown whether surgical intervention in the present situation, which involves complete paralysis of the lower extremity and the loss of reflexes, improves the neurological prognosis of the lower extremity. In this case, early intervention on the affected side would unlikely improve neurological abnormalities at birth due to the absence of neurologic response. In contrast, clinicians should control the progression of neurological abnormalities on the healthy side with surgical intervention owing to the possible progression of the tethered cord syndrome in the future. There is insufficient evidence available to determine the appropriate timing of surgical intervention, thus warranting further investigation.

## 4. Conclusions

Herein, we reported on a case of type II SCM with hypoplasia and paralysis of the left lower extremity, along with a cutaneous dimple of the lumbosacral region. However, early intervention may improve its neurological prognosis, thus necessitating a thorough examination of the entire spine in cases of left–right differences in the lower extremities with skin abnormalities in the lumbosacral region.

## Data Availability

The data that support the findings of this study are not publicly available due to their containing information that could compromise the privacy of research participants but are available from the corresponding author [T.I.] upon reasonable request.
